# Innervation of Gonadotropin-Releasing Hormone Neurons by Peptidergic Neurons Conveying Circadian or Energy Balance Information in the Mouse

**DOI:** 10.1371/journal.pone.0005322

**Published:** 2009-04-24

**Authors:** Daniel R. Ward, Fiona M. Dear, Ian A. Ward, Susan I. Anderson, Daniel J. Spergel, Paul A. Smith, Francis J. P. Ebling

**Affiliations:** 1 School of Biomedical Sciences and Institute for Neuroscience, University of Nottingham Medical School, Nottingham, United Kingdom; 2 Section of Endocrinology, Department of Medicine, University of Chicago, Chicago, Illinois, United States of America; Pennsylvania State University, United States of America

## Abstract

**Background:**

Secretion of gonadotropin-releasing hormone (GnRH) produced in neurons in the basal forebrain is the primary regulator of reproductive maturation and function in mammals. Peptidergic signals relating to circadian timing and energy balance are an important influence on the reproductive axis. The aim of this study was to investigate the innervation of GnRH neurons by peptidergic neurons.

**Methodology/Principal Findings:**

Immunohistochemistry and confocal microscopy were used to detect appositions of peptidergic fibers (NPY, β-endorphin, MCH) associated with energy balance and metabolic status in transgenic mice expressing a green fluorescent protein reporter construct in GnRH neurons. The frequency of these appositions was compared to those of vasoactive intestinal peptide (VIP), a hypothalamic neuropeptide likely to convey circadian timing information to the GnRH secretory system. The majority of GnRH neurons (73–87%) were closely apposed by fibers expressing NPY, β-endorphin, or MCH, and a significant proportion of GnRH neurons (28%) also had close contacts with VIP-ir fibers.

**Conclusions/Significance:**

It is concluded that GnRH neurons in the mouse receive a high frequency of direct modulatory inputs from multiple hypothalamic peptide systems known to be important in conveying circadian information and signalling energy balance.

## Introduction

Gonadotropin-releasing hormone-1 decapeptide (henceforth referred to as GnRH) is the final common signalling molecule in the brain that regulates reproductive function in all vertebrates [1]. The GnRH secretory system appears to have an extraordinarily large array of modulatory inputs conveying information about the internal (e.g. steroids, nutritional status) and the external (e.g. photoperiod, olfactory cues) environment [2]. The neuroanatomical and functional characterization of the pathways that transduce these inputs to the GnRH secretory system has in the past been hampered by the diffuse distribution and relative paucity of GnRH neurons in the basal forebrain [3]. However, the development of transgenic mice in which a GnRH promoter drives a fluorescent reporter construct has provided a valuable tool to aid the investigation of GnRH neurobiology [4], [5]. Using this strategy to identify individual GnRH neurons in combination with RT-PCR, Todman et al [6] recently reported that GnRH neurons express mRNAs encoding at least 50 different receptors for neurotransmitters or neuromodulators, including 14 different neuropeptide receptor families.

In mammals, reproduction is one of the most energy costly processes, hence it is imperative that the mechanisms controlling energy balance integrate with those that control reproduction [7]. Given the importance of multiple hypothalamic peptidergic systems in the homeostatic control of energy balance, it seems likely that they play a major role in signalling to GnRH neurons [8]. There is functional evidence for a number of such peptides in the control of GnRH secretion. For example, β-endorphin, a major product of the pro-opiomelanocortin gene which colocalizes with the anorectic peptide αMSH in the arcuate nucleus [9], inhibits GnRH secretion [10]. In contrast, the orexigenic peptides neuropeptide Y (NPY) and melanin-concentrating hormone (MCH) have steroid-dependent stimulatory and inhibitory effects on the secretion of LH [11]–[13]. It remains to be determined whether the effects of such peptides are exerted directly upon GnRH neurons, or indirectly via intermediary neurons. For example, it is controversial as to whether GnRH neurons express the μ-opioid receptor and thus whether they are directly regulated by β-endorphin [14]. The aim of the present study was therefore to use single-label immunohistochemistry in transgenic mice expressing a GnRH promoter-GFP (green fluorescent protein) reporter construct combined with fluorescent and confocal microscopy to determine whether neuroanatomical inputs from NPY-, β-endorphin-, and MCH- containing fibers form close appositions with GnRH neurons and are therefore likely to regulate GnRH secretion directly. The incidence of these peptidergic inputs was compared to that of vasoactive intestinal peptide (VIP) -ergic appositions because extensive evidence exists that this peptide conveys circadian information from the SCN to the GnRH secretory system and thereby regulates the timing of the proestrous LH surge in rodents [15]–[17].

## Materials and Methods

### Animals

Adult male and randomly-cycling female GnRH-GFP mice (B6/GnRHhGFP2) [4] were obtained from a breeding colony at the University of Nottingham maintained on a 12:12h light/dark cycle with ambient temperature at 21±1°C, and had access to food and water *ad libitum*. Studies were approved by the University of Nottingham Local Ethical Review Committee and conducted in accordance with the United Kingdom Animals (Scientific Procedures) Act, 1986.

### Immunohistochemistry

GnRH-GFP mice were terminally anesthetized with Euthatal (sodium pentobarbitone, Rhone Merieux, Harlow, UK). The chest cavity was opened, and 500 units of heparin (CP Pharmaceuticals Ltd, Wrexham, UK) were injected intracardially. Mice were then perfused via gravity flow through the ascending aorta with PBS (0.01 M phosphate buffered 0.9% saline, pH 7.2) for 3 minutes followed by 4% paraformaldehyde (PFA in 0.1 M phosphate buffer, pH 7.2) for 5 minutes. Brains were dissected out and placed in 4% PFA for 3–4 hours then cryopreserved by overnight incubation in 20% sucrose in PBS. Brains were sectioned in the coronal plane on a freezing microtome at a thickness of 60 µm.

Immunohistochemical procedures were carried out on free-floating sections at room temperature. For NPY and MCH antisera, sections were placed into small jars of citrate buffer (10 mM, pH6.0) and placed in a 650 W microwave on high power for ∼12 seconds to promote antigen retrieval prior to staining. Sections were allowed to cool for 10 minutes and washed twice in PBS. Sections were incubated for 1 hour in 2% normal goat serum (in buffer 1; PBS containing 1% BSA, 0.3% Triton X-100), then in primary antisera (in buffer 1) overnight at 4°C. Additional sections were incubated in normal rabbit serum or no serum as control procedures. Primary antisera were i) rabbit polyclonal anti-GnRH antiserum which recognizes amino acids 6–10 of GnRH in pro-GnRH and GnRH (LR1; a kind gift from Dr Robert Benoit, McGill University), used at a final dilution of 1∶15,000, ii) rabbit polyclonal anti-VIP raised against N-terminal of rat VIP peptide (a kind gift from Drs Jens Mikkelsen and Jan Fahrenkrug; University of Copenhagen, [18]) used at a final dilution of 1∶5,000, iii) rabbit polyclonal anti-NPY, kindly donated by Prof Julia Polak, Imperial College London, [19] used at a final dilution of 1∶2000, iv) rabbit polyclonal anti-rat β-endorphin[1]–[31], kindly donated by Dr Eric Bittman, University of Massachusetts, [20] used at a final dilution of 1∶1,000, v) rabbit anti-rat/human synthetic MCH, kindly donated by Dr Bridget Baker, University of Bath, [12] used at a final dilution of 1∶1,000.

After 3 washes in buffer 2 (PBS containing 0.3% BSA and 0.1% Triton X-100) sections were incubated in biotinylated goat anti-rabbit IgG (Vector Labs, Peterborough, UK) at 1∶400 (in buffer 2) for 1 hour at room temperature. Following a further 2 washes in buffer 2, and 1 wash in PBS, sections were incubated with avidin-TRITC conjugate (tetramethyl rhodamine iso-thiocyanate, Vector) at 1∶200 (in buffer 2). Sections were then washed again; twice in buffer 2 and once in PBS prior to mounting on gelatin-subbed slides and air-drying. Slides were briefly washed in distilled H_2_O prior to coverslipping with glycerol.

### Fluorescence microscopy

Sections were initially viewed under a Leica DMRB fluorescence microscope to identify the locations of GnRH-GFP neurons and immunostained peptidergic perikarya and fibers. Sections were examined using filters appropriate for visualisation of GFP and TRITC, and images were captured and superimposed using Openlab software (Improvision, Coventry, UK). Each GnRH-GFP cell soma was scored by a single investigator for the presence and number of ‘close appositions’, defined as immunostained peptide fibers or putative terminals which appeared to either directly contact the GnRH-GFP cell or which overlapped the cell and were in the same plane of focus.

### Confocal microscopy

To confirm that the overlap or contact of peptide-immunostained structures with GnRH-GFP cell soma and dendrites identified using fluorescence microscopy truly represented close contacts, sections with putative appositions of each peptide were examined further using a confocal laser scanning microscope (Leica SP2), with a Leica IRE2 inverted fluorescence microscope to help relocate GnRH-GFP neurons. GFP was excited using the 488 nm argon laser line (emission 500–550 nm) and TRITC-labelled structures were sequentially excited (to avoid cross-talk between the wavelengths during acquisition) using the 561 nm helium-neon laser line (emission 570–620 nm). Each cell was examined through its entirety in 0.5 µm steps (∼20 slices) and all appositions were assessed. Images were displayed as maximum intensity projections of the entire z-series or as single 0.5 µm slices using the associated Leica LCS software.

## Results

### Validation of GnRH-GFP mice

**Figure 1:**
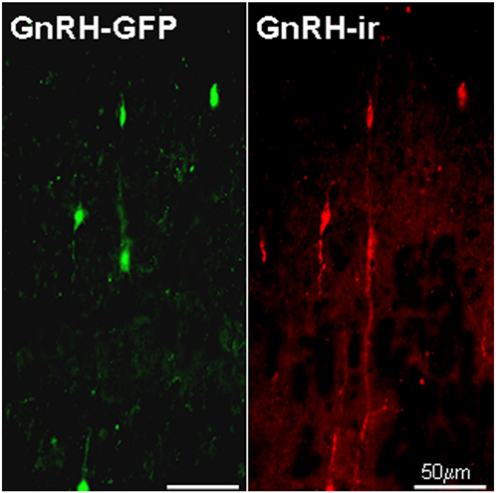
Confocal microscopy revealing GFP-expressing GnRH neurons (left) and immunostaining with LR1 (right). Note that the two techniques reveal a similar number of GnRH perikarya, but the extent of the neurites is clearer using the immunohistochemical approach. Scale bars = 50 µm.

In order to confirm that the distribution of GFP-expressing neurons accurately represents the distribution of GnRH positive neurons, a series of sections from 2 transgenic adult male mice were immunostained with an anti-GnRH antibody (LR1) using a TRITC fluorescent tag. There was a very high incidence of colocalization of GnRH immunoreactivity and GFP (Fig. 1). Of the 140 GnRH-immunopositive perikarya that were analyzed, 132 (94%) were also GFP-positive. Conversely all GFP-positive neurons were LR1-positive. Using fluorescence microscopy, the extent of the GnRH neurites appeared to be greater with immunostaining than revealed by the GFP signal (Fig. 1).

### Appositions between VIP-ir axons and GnRH neurons

**Figure 2:**
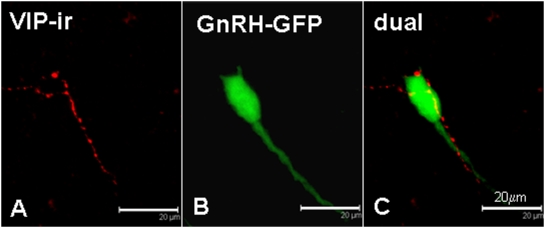
VIP-GnRH interactions. Confocal microscopy photomicrographs of 0.5 µm optical sections showing the relationship between a vasoactive intestinal polypeptide fiber (A: VIP, red) and a GnRH-GFP perikaryon (B: GnRH, green). Panel C shows the superimposed images. Scale bars = 20 µm.

VIP-ir varicose fibers and terminals were scattered throughout the GnRH continuum. Of 510 GFP-positive neurons examined in sections from 3 female mice, 28% were contacted by VIPergic axons (Fig. 2, Table 1). Some VIPergic appositions to GnRH perikarya located in the anterior hypothalamus were traceable through the coronal plane of the section to their origin, all of which were located in the SCN. When the proportion of GnRH neurons contacted by VIPergic terminals was investigated according to brain region it was found that those in the rostral (medial septum and diagonal band of Broca) and medial (preoptic area) aspects of the GnRH continuum received a roughly similar proportion of VIP appositions: 30±1% and 32±4% respectively. However, in more caudal regions (anterior and medial hypothalamus) where a small proportion of GFP-GnRH neurons were detected these neurons had significantly fewer close appositions from VIP-ir fibers (19±1%; *p*<0.01 vs rostral areas, χ^2^ test).

**Table 1:** Summary of peptidergic appositions on GnRH-GFP perikarya.

peptide	# mice	# GnRH-GFP cells analyzed	0 appositions	≥1 apposition	% ≥1 apposition
VIP	3	510	370	140	28%
NPY	4	52	7	45	87%
β-endorphin	4	71	19	52	73%
MCH	4	58	8	50	86%

Analysis of close appositions between peptidergic fibers and GnRH-GFP perikarya using fluorescence microscopy.

### Appositions between NPY-ir axons and GnRH neurons

**Figure 3:**
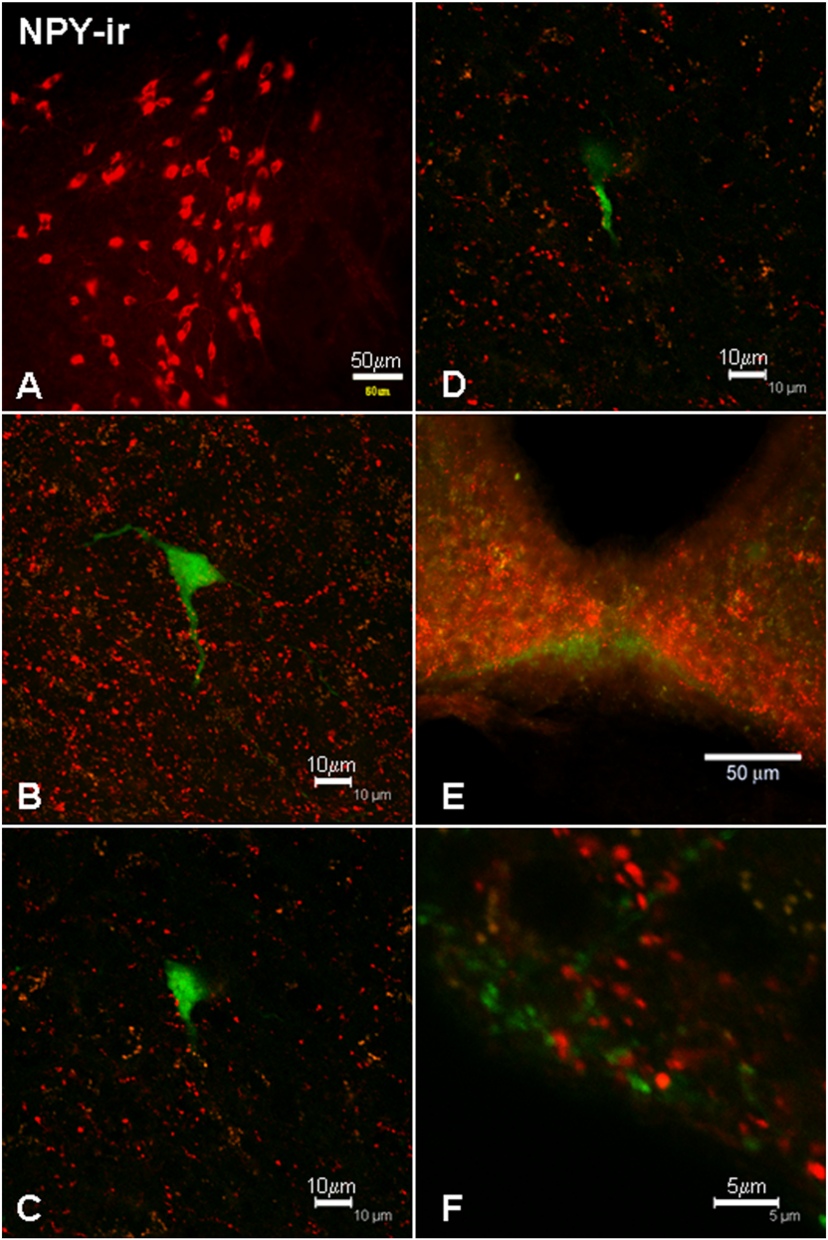
A: NPY-ir perikarya localized in the arcuate nucleus. Examples of close appositions between NPY-ir fibers (red) and GnRH-GFP perikarya and processes (green) in a maximum intensity projection of the image stack (B) and in single 0.5 µm optical slices (C, D). NPY-ir fibers and GnRH-ir terminals were observed in proximity in the median eminence (E), where the presence of close appositions was confirmed by analysis of single 0.5 µm optical slices (F). Scale bars = 50 µm (A), 10 µm (B–D), 50 µm (E), 5 µm (F).

NPY-ir perikarya within the hypothalamus were restricted to the arcuate nucleus (Fig. 3A), but there was an extensive distribution of NPY-ir fibers and terminals throughout the basal forebrain, including all regions where GnRH perikarya were located. Out of 52 GnRH neurons examined (in sections from 4 male mice), 87% displayed close appositions with NPY-ir fibers (Fig. 3B–D, Table 1). The extensive innervation of the medial septum by NPY-ir fibers is clearly apparent (Fig. 3B). The proportions of GnRH neurons receiving close appositions from NPY-ir fibers were similar across the basal forebrain: medial septum/diagonal band of Broca (MS/DBB) 84%; anterior hypothalamus 100%; and mediobasal hypothalamus 100%. There was no statistically significant difference in the proportion of GnRH neurons with appositions between these areas (*p* = 0.38, χ^2^ test). The mean number of appositions per GnRH-GFP neuron was 2.45, and there was no statistical difference in the number of NPY appositions per neuron between regions (*p* = 0.45, Median test). There was some overlap between NPY-ir fibers and GnRH neuroterminals in the caudal median eminence (Fig. 3E). Although NPY-ir fibers were predominantly localized in the internal layer and GnRH fibers in the external layer (Fig. 3E), close appositions were confirmed by the analysis of single 0.5 µm optical sections (Fig. 3F).

### Appositions between β-endorphin-ir axons and GnRH neurons

**Figure 4:**
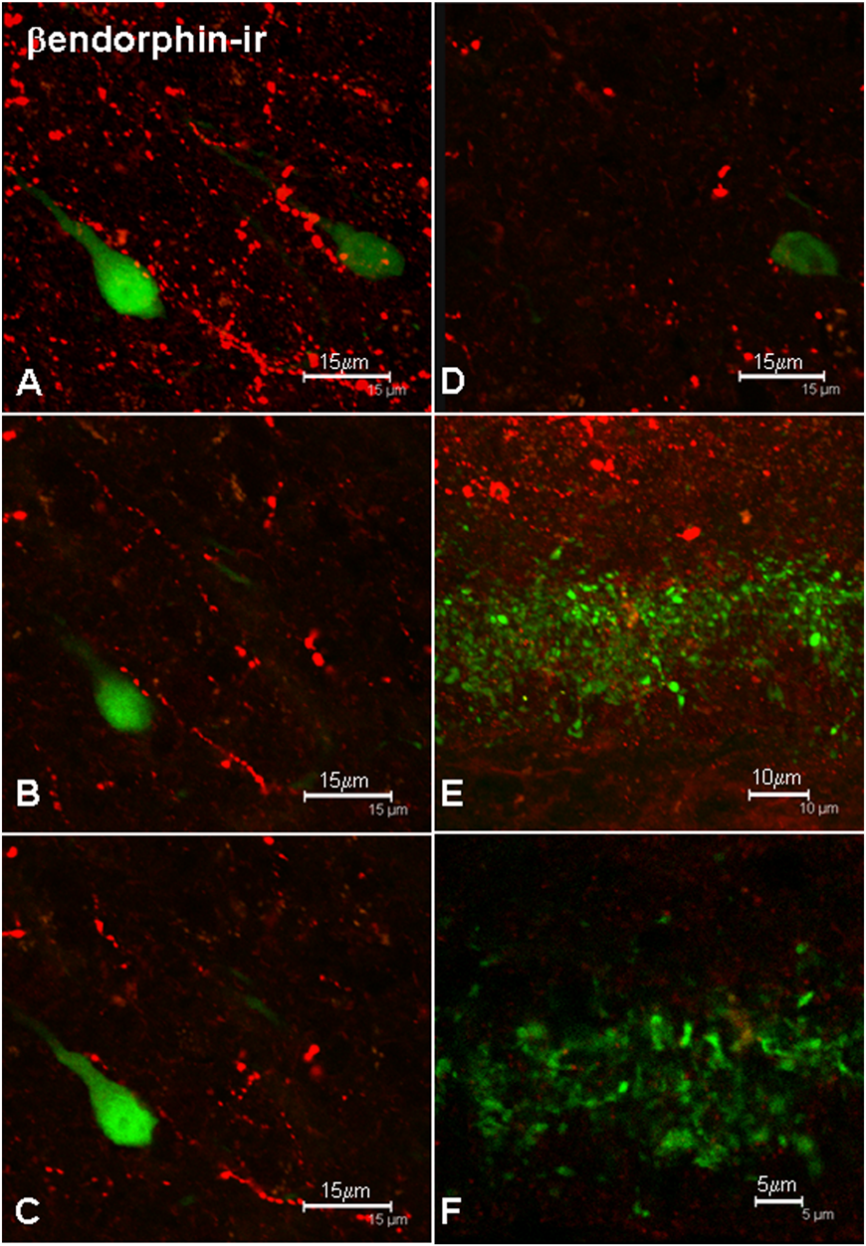
Examples of close appositions between β-endorphin-ir fibers (red) and GnRH-GFP perikarya and processes (green) in a maximum intensity projection of the image stack (A) and in single 0.5 µm optical slices (B–D). A few close appositions were also observed between β-endorphin-ir fibers and GnRH neuroterminals in the median eminence (E), confirmed by analysis of single 0.5 µm optical slices (F). Scale bars = 15 µm (A–D), 10 µm (E), 5 µm (F).

β-endorphin-ir perikarya were restricted to the arcuate nucleus, but immunoreactive fibers innervated all areas where GnRH perikarya were located (e.g., medial septum, Fig. 4A) and made close appositions with GnRH-GFP neurons in all brain sections examined (Fig 4A–D). Of a total of 72 GnRH perikarya examined from 4 male mice, 73% received close appositions from β-endorphin-ir fibers (Table 1). Analysis of confocal image stacks of optical sections confirmed the presence of close appositions between β-endorphin-ir fibers and GnRH neurons (Figs. 4B–D). The proportion of GnRH perikarya receiving close appositions from β-endorphin-ir fibers was similar across regions of the basal forebrain: 80% of perikarya neurons in the region of the MS/DBB received close contacts from β-endorphin-ir fibers, 62% in the anterior hypothalamus and 60% in the mediobasal hypothalamus (*p* = 0.22, χ^2^ test). β-endorphin-ir fibers were also apposed to GnRH neuroterminals in the external layer of the median eminence (Fig. 4E). The presence of close appositions between these two types of fiber was confirmed by the analysis of single 0.5 µm optical sections (Fig. 4F).

### Appositions between MCH-ir axons and GnRH neurons

**Figure 5:**
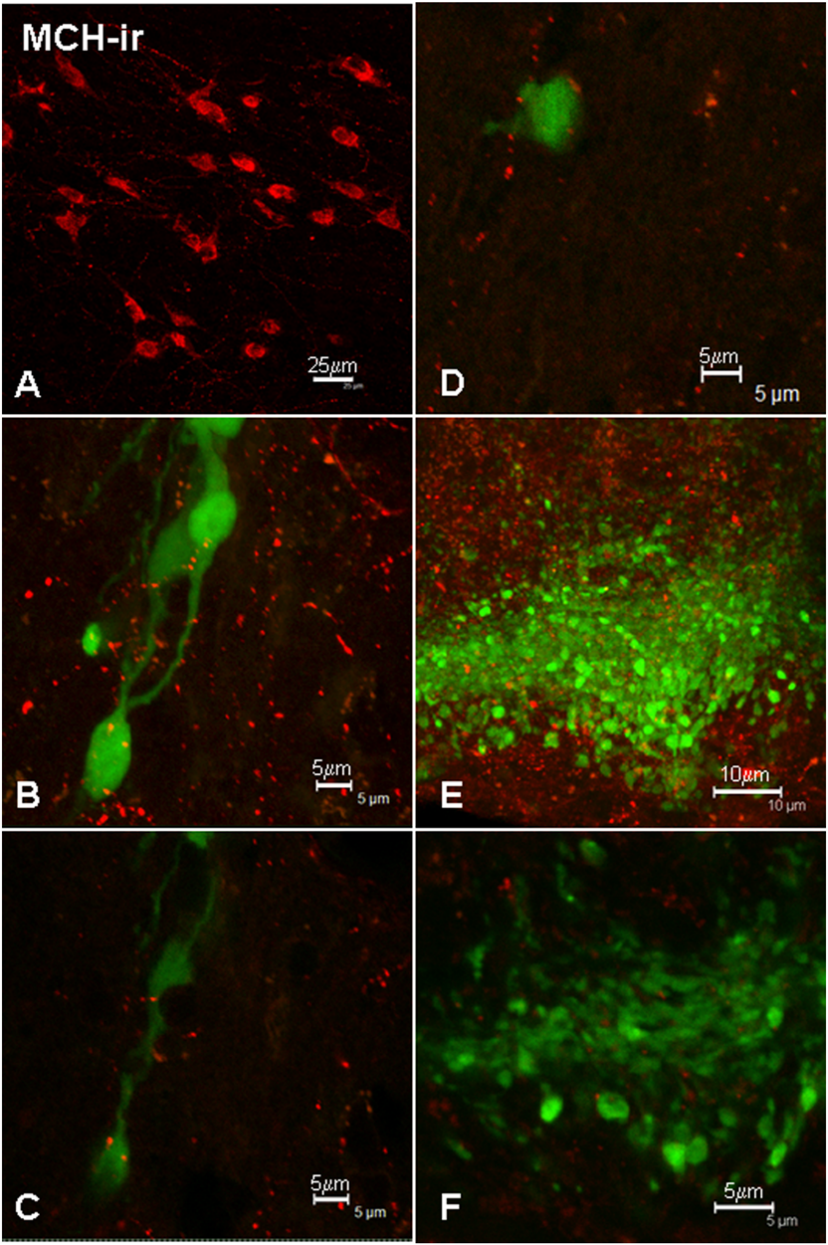
A: MCH-ir perikarya localized in the lateral hypothalamic area (A). Examples of close appositions between MCH-ir fibers (red) and GnRH-GFP perikarya and processes (green) in a maximum intensity projection of the image stack (B) and in single 0.5 µm optical slices (C, D). MCH -ir fibers and GnRH-ir terminals were observed in proximity in the median eminence (E), where the presence of close appositions was confirmed by analysis of single 0.5 µm optical slices (F). Scale bars = 25 µm (A), 5 µm (B–D), 10 µm (E), 5 µm (F).

MCH-ir perikarya were widely distributed in the lateral hypothalamus of the mouse (Fig. 5A), and MCH-ir fibers innervated many forebrain areas (e.g., Fig. 5B). Close appositions between MCH-ir fibers and GnRH-GFP perikarya were observed in the medial septum and all other brain areas examined (Fig. 5B). The close proximity of appositions between GnRH perikarya and MCH-ir fibers was confirmed by the analysis of single 0.5 µm optical slices (Fig. 5B–D). Of a total of 60 GnRH neurons examined from 4 male mice, 86% showed close appositions with MCH-ir fibers (Table 1). The proportion of GnRH neurons receiving close appositions from MCH-ir fibers was similar in all regions examined: MS/DBB 87%; anterior hypothalamus 86%; and mediobasal hypothalamus 88%, and there was no statistically significant difference (*p* = 0.58, Median test). In addition, there was some overlap between MCH-ir fibers and GnRH neuroterminals in the external layer of the ME (Fig. 5E). The presence of close appositions between these two types of fiber was confirmed by the analysis of single 0.5 µm optical sections (Fig. 5F).

## Discussion

Confocal microscopy proved to be a highly sensitive method for detecting GFP-positive GnRH perikarya because the vast majority (94%) of immunohistochemically labelled GnRH neurons also expressed GFP. In the same strain of transgenic mice and using the same antiserum to identify GnRH neurons, Spergel et al. originally observed GFP fluorescence in only 65% of GnRH immunoreactive neurons [4] indicating reduced fading of GFP and/or improved sensitivity of GFP detection with our current methodology. The close correspondence between immunohistochemical identification of GnRH neurons and GFP expression also suggests that despite using relatively thick sections (60 µm), the tissue processing prior to immunostaining involving antigen retrieval in citrate buffer and use of triton detergent in the buffers was sufficient to ensure good penetration of antibodies throughout the whole thickness of the tissue section.

A significant body of evidence implicates VIP in the regulation of the preovulatory LH surge in rodents [21]. In agreement with previous studies in female rats, we found frequent VIPergic appositions on GnRH neurons in the female mouse. Horvath et al. [22] and Kreigsfeld et al. [23] reported that 35% and 5% respectively of GnRH neurons in the rat were contacted by VIPergic structures. The figure obtained in the current murine study (28%) is clearly closer to the former, and is consistent with the proportion of GnRH neurons in the rat co-expressing VIP receptors (VPAC2; ∼40%;[24]). Horvath et al. [22] also found that destruction of the SCN caused an 80% decrease in VIP-GnRH appositions in rat, suggesting a role for VIP in communicating circadian information from the SCN to the GnRH secretory system. Thus, our observations of VIP-ir appositions to GnRH-GFP neurons indicate that our experimental and analytical procedures using GnRH-GFP transgenic mice possess sufficient sensitivity to replicate established dual-label immunohistochemical procedures. We recognise that the precise anatomical structure of close appositions cannot be resolved without electron microscopy analysis, however a recent ultrastructural study which imaged close appositions of galanin-like peptide-immunostained fibers on GnRH-GFP cell soma in the rat found extensive evidence that the peptidergic appositions were indeed synaptic in nature [25]. A dual-immunolabel ultrastructural study in the pigeon also found evidence that close appositions of VIPergic fibers on GnRH-immunoreactive cell soma were truly direct contacts between VIPergic fibers and GnRH neurons, however the contacts lacked synaptic specialisations such as a thickened membrane [26]. In addition, some of the close appositions of VIP-ir fibers and GnRH neurons in the preoptic area of the rat may actually represent VIPergic input to astrocyte processes that ensheath GnRH neurons [27].

Three peptidergic families known to be of considerable importance in the central control of energy balance, NPY [28], β-endorphin/α-MSH [29], and MCH [30], all displayed numerous close appositions with GnRH perikarya in the rostral forebrain, and a higher proportion of GnRH neurons received these appositions than received VIP-ir contacts. 87% of GnRH neurons examined received close appositions from NPY-ir fibers, consistent with previous studies in the rat [31] and sheep [32]. Triple-label immunofluorescence studies in the rat suggest the effects of NPY on GnRH perikarya might be mediated by the Y5 receptor subtype [31]. We also observed close appositions between NPY and GnRH fibers in the median eminence, supporting the likelihood that NPY regulates GnRH secretion at the level of GnRH neuroterminals in this structure. This is consistent with previous findings in the rat, which suggest NPY may be directly involved in modulating GnRH secretion via Y1 receptors located on GnRH neuroterminals in the median eminence [33]. NPY has been considered to be one of the most potent orexigenic peptides in the brain. NPY neurons in the arcuate nucleus express leptin receptors [34], making NPY a strong candidate for conveying information on fat reserves to the reproductive axis. In addition, it is likely that NPYergic fibers convey information to GnRH neurons from the brainstem and other areas since a previous study in mice found that neonatal ablation of the arcuate nucleus decreased NPYergic appositions to GnRH perikarya and proximal dendrites by approximately half [35].

The distribution of β-endorphin-ir fibers in the basal forebrain and the localization of β-endorphin-ir perikarya within the arcuate nucleus of the mouse that we report here are consistent with previous findings in the rat [36]. A high proportion of the pro-opiomelanocortin-expressing neurons that give rise to β-endorphin in the arcuate nucleus express leptin receptors [34], and these cells in the arcuate nucleus all express αMSH [9] which is generally considered to be the more important POMC product in terms of regulation of food intake and energy expenditure. Thus, our demonstration of close appositions between β-endorphin-ir fibers and GnRH perikarya is consistent with the view that this is an inhibitory mechanism by which information about systemic fat stores (leptin) reaches the GnRH secretory system.

The distribution of MCH-ir fibers in the basal forebrain and the localization of MCH-ir perikarya in the lateral hypothalamic area and zona incerta in the mouse are also consistent with previous reports in the rat [37]. The results from our confocal analysis suggest the connections between the MCH signalling system and GnRH neurons are very abundant, with 86% of GnRH perikarya receiving close appositions from MCH-ir fibers. These findings are consistent with a recent dual-label immunohistochemistry study in the rat, which reported that 80–90% of GnRH perikarya in the medial preoptic area and anterior hypothalamus had close appositions from MCH-ir fibers [38]. Several lines of evidence support a role for MCH as a potent orexigen and, like many peptides involved in energy homeostasis, MCH has been proposed to link energy status to reproductive function. The effects of MCH on GnRH release appear to be estrogen-dependent: MCH inhibits the secretion of LH in the presence of low estrogen [13], whilst a stimulatory action has been reported under conditions of high estrogen [12]. The current anatomical evidence suggesting functional interactions between MCH and GnRH is also supported by evidence of MCH receptor expression. GT1-7 cells have been shown to express MCH-R1 mRNA [39], and it has been recently reported that 50–55% of GnRH neurons express MCH-R1 mRNA in the rat, suggesting MCH-R1 receptors may be responsible for mediating the effects of MCH on GnRH neurons [38]. In addition, our results provide evidence to support the hypothesis that MCH can influence GnRH secretion at the level of GnRH neuroterminals in the median eminence. MCH fibers were present in the median eminence, consistent with previous findings [37], [38]. Our analysis of individual 0.5 µm optical sections using confocal microscopy demonstrated the presence of close appositions between MCH-ir fibers and GnRH neuroterminals in the median eminence, consistent with previous findings in the rat [38].

This neuroanatomical circuitry revealed in the mouse in the current study is likely to be of functional significance. It is clear that there is a circadian rhythm of VIP abundance in the mouse SCN [40], and electrophysiological studies have recently demonstrated that in the presence of estrogen, VIP directly excites GFP-flagged GnRH neurons in hypothalamic slices [21]. Moreover our observation that 28% of GnRH neurons received close VIPergic appositions is consistent with their observations that between 17% and 46% of GnRH-GFP were excited by VIP dependent upon whether the tissues were taken from the mice during a negative feedback phase or during an estrogen-induced LH surge [21]. It is also clear that the expression of genes encoding POMC, NPY and MCH in the hypothalamus is regulated by energy balance in the mouse [41], [42], and that the products of these genes affect GnRH/LH secretion. β-endorphin has been shown to inhibit LH secretion in a wide variety of species [43], and specifically in mice it has been shown that blockade of μ-opioid receptors with naloxone potentiates LH release, for example when induced by glutamatergic agonists [44]. Likewise, there are a number of studies in rats [45], [46] which demonstrate that blockade of NPY receptors with antagonists elicits LH release. Evidence for a direct functional pathway between MCH fibers and GnRH neuroterminals in the median eminence is supported by *ex vivo* experiments, which showed the addition of MCH to explants of the ventral hypothalamus from proestrous rats increased GnRH release [47].

### Conclusions

The current studies reveal that GnRH neurons in the mouse receive a high frequency of close appositions from peptidergic systems known to be important in conveying circadian information (VIP) and signalling energy balance (NPY, β-endorphin and MCH). The current study adds to the evidence that GnRH perikarya and terminals have a plethora of different peptidergic inputs, as other studies have generated either neuroanatomical or functional evidence that a wide range of other peptides also regulate GnRH secretion (e.g. CART, kisspeptin, orexin, prokineticin). This huge diversity of peptidergic inputs raises the question of their individual significance. The complete infertility in genetically modified mice lacking kisspeptin [48] and its cognate receptor GPR54 [49], or lacking prokineticin 2 [50] or prokineticin receptor 2 [51] suggests that some peptidergic systems are of critical importance in hypothalamic control of fertility. However the majority of metabolic peptidergic systems appear to play a redundant role, in that although pharmacological manipulation of such peptides affects gonadotropin secretion, the genetic ablation of most peptides or their cognate receptors implicated in metabolic or circadian control does not produce infertility e.g. NPY [52], MCH [53], orexin [54], melanocortin receptor 4 [55], pro-opiomelanocortin [56] and VIP [57]. Integrated modulation by multiple peptides, as opposed to the highly organised structure of cortical systems, appears to be a general feature of the limbic system [58], so the recent attempts to apply systems biology methodologies to advance our understanding of the multivariate control of GnRH secretion are increasingly important [59].
